# Metamaterial-enhanced vibrational absorption spectroscopy for the detection of protein molecules

**DOI:** 10.1038/srep32123

**Published:** 2016-08-24

**Authors:** Tung S. Bui, Thang D. Dao, Luu H. Dang, Lam D. Vu, Akihiko Ohi, Toshihide Nabatame, YoungPak Lee, Tadaaki Nagao, Chung V. Hoang

**Affiliations:** 1 Institute of Materials Science (IMS), Vietnam Academy of Science and Technology (VAST), 18 Hoang Quoc Viet Street, Cau Giay District, Hanoi, Vietnam; 2 Quantum Photonic Science Research Center, Department of Physics, Hanyang University, Seoul 133-791, Korea; 3 International Center for Materials NanoArchitectonics (MANA), National Institute for Materials Science (NIMS), 1-1 Namiki, Tsukuba 305-0044, Japan; 4 CREST, Japan Science and Technology Agency (JST), Kawaguchi, Saitama 332-0012, Japan; 5 Department of Condensed Matter Physics, Graduate School of Science, Hokkaido University, Kita-10 Nishi-8 Kita-ku, Sapporo 060-0810, Japan

## Abstract

From visible to mid-infrared frequencies, molecular sensing has been a major successful application of plasmonics because of the enormous enhancement of the surface electromagnetic nearfield associated with the induced collective motion of surface free carriers excited by the probe light. However, in the lower-energy terahertz (THz) region, sensing by detecting molecular vibrations is still challenging because of low sensitivity, complicated spectral features, and relatively little accumulated knowledge of molecules. Here, we report the use of a micron-scale thin-slab metamaterial (MM) architecture, which functions as an amplifier for enhancing the absorption signal of the THz vibration of an ultrathin adsorbed layer of large organic molecules. We examined bovine serum albumin (BSA) as a prototype large protein molecule and Rhodamine 6G (Rh6G) and 3,3′-diethylthiatricarbocyanine iodide (DTTCI) as examples of small molecules. Among them, our MM significantly magnified only the signal strength of bulky BSA. On the other hand, DTTCI and Rh6G are inactive, as they lack low-frequency vibrational modes in this frequency region. The results obtained here clearly demonstrate the promise of MM-enhanced absorption spectroscopy in the THz region for detection and structural monitoring of large biomolecules such as proteins or pathogenic enzymes.

Among plasmonic sensing techniques, surface-enhanced vibrational spectroscopy (SEVS), which includes surface-enhanced Raman scattering (SERS)^1^,^2^,^3^ and surface-enhanced infrared absorption spectroscopy (SEIRA)^4^,^5^,^6^, has been widely studied and has proven to be one of the most successful applications of plasmonics to date. The advantage of SEVS is the use of a huge electromagnetic field enhancement at the surface of plasmonic nano-objects originating from ultrafast oscillatory motion of the high-density electron gas excited at metal surfaces^5^,^6^. For example, in SEIRA, the oscillating electromagnetic field of the vibrating molecules interacts strongly with (interferes with or scatters from) the plasmonically enhanced nearfield, thus providing strong enhancement of the light absorption at their vibrational frequencies, which consequently yields ultrahigh sensitivity^6^,^7^ that routinely surpasses the single monolayer level.

In the terahertz (THz) region, metal is considered an ideal conductor with nearly unity reflectivity and limited skin depth^8^. The ratio of the skin depth and wavelength of the impinging radiation in free space in the THz region is much smaller than that in the optical range. Thus, it is more difficult to focus and guide THz surface electromagnetic waves, or surface polaritonic waves, more than waves at optical frequencies. However, THz surface waves can travel without being significantly absorbed in many materials, so they have some advantageous features for spectroscopic as well as imaging device applications^8^,^9^. For example, molecular sensing in the THz region has become a key approach and has been widely studied^10^,^11^,^12^,^13^,^14^. A common method using a metamaterial (MM) is dielectric surface plasmon resonance sensing in the THz region, where the presence of molecules can be detected by the spectral shift caused by absorption of the molecules on the device^15^,^16^,^17^,^18^,^19^,^20^. Sensing with a split-ring resonator (SRR) is a typical example^9^,^10^,^13^,^15^,^16^,^17^,^21^,^22^. This structure is composed of a tiny gap area, whose resonant frequency can be well described by an equivalent LC circuit. Because the presence of trace molecules deposited on the SRR induces a change in the resonant frequency, the quantity of deposited molecules can be sensitively detected. This method obviously offers a sensitive approach to molecular detection, but it does not offer high selectivity for organic molecules because the dynamic range of the variation in their dielectric constant is narrow (approximately 3–4), which limits application of the method.

In this paper, we examine the applicability of MM-enhanced absorption spectroscopy for molecular sensing in the THz domain. Like SEIRA, this method relies on the enhancement of vibrational signals of the absorbed molecules; here it is realized by matching the vibrational frequency of target molecules to the resonant frequency of rationally designed MMs. An ultrathin layer of bovine serum albumin (BSA) molecules deposited on the MM is detected with a signal strength almost comparable to those of submicron-thick bulky materials owing to the strong field enhancement of the resonant MM. For small organic molecules of other materials that do not exhibit any distinct signals in the THz region, no enhanced signals are obtained. This clearly shows the selectivity of this method for the detection of large protein molecules, the vibrational fingerprints of which appear mainly in the THz region. Our results demonstrate the use of THz vibrational sensing for large biomolecules and offer an opportunity to widen the application of THz MMs.

## Results

### Sample design

**Figure 1:**
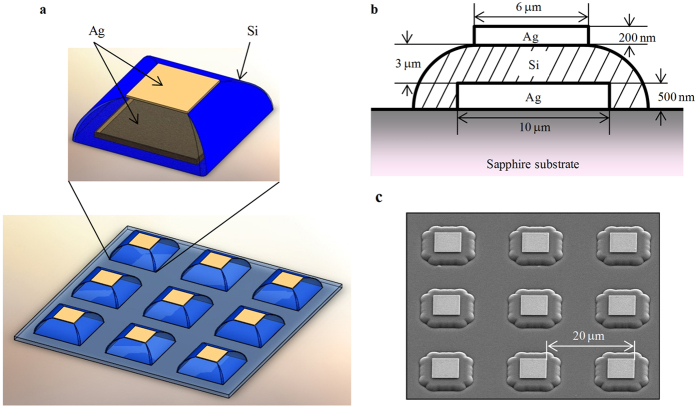
(**a**) Schematic illustrations of the MM sample in this study. (**b**) Cross-sectional illustration of the sample design with detailed dimensions of the sample. (**c**) SEM image of a typical sample. Small steps at the corners of the samples were mistakenly created during fabrication.

Our proposed Ag–Si–Ag trilayered MM structure is shown in Fig. 1a,b. Figure 1c shows a 30°-tiled-view scanning electron microscope (SEM) image of the fabricated MM device. Two Ag disk arrays, used as back and top resonators that sandwich a Si insulator, were placed on a sapphire substrate. The geometrical parameters of the MM structure were optimized using an electromagnetic simulation. Here the MM is aimed at a dual-band resonance at approximately 5 THz, which resonates with the absorption signal of the targeted BSA molecules. Different thicknesses (0.2 and 0.5 μm) and different widths (10 and 6 μm) were chosen for the top and bottom Ag disk resonators, respectively. The thickness of the Si insulator and the periodicity were 3 and 20 μm, respectively.

### Optical properties

**Figure 2:**
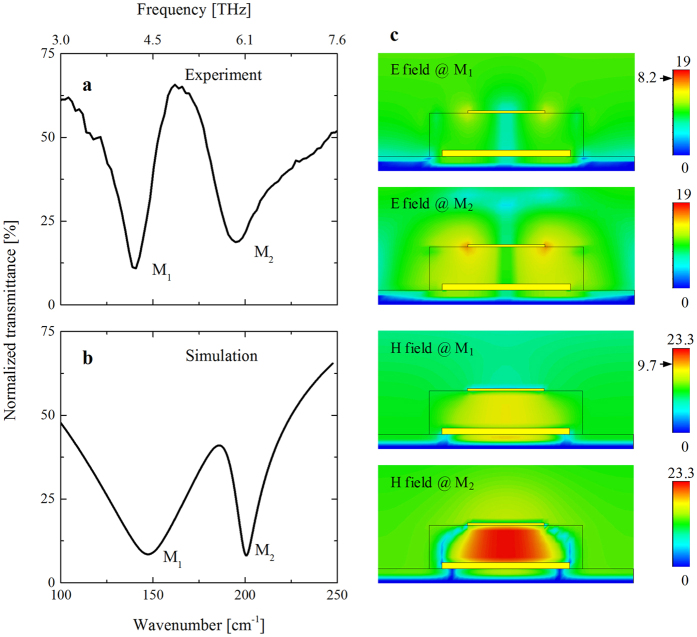
(**a**) Measured and (**b**) simulated transmittance spectra of the MM structure. There were two resonant peaks, M1 (at low frequency) and M2 (at high frequency), which were related to the photonic–magnetic dipole coupling and magnetic resonances, respectively. For details, see the text. (**c**) Simulated electric and magnetic field distributions at the MM structure with excitations in the low-frequency (M1) and high-frequency (M2) modes. Color scale bars in (**c**) show the enhanced electric and magnetic fields compared to the incident fields; arrows indicate the maximum field enhancements for low-frequency (M1) excitation.

Figures 2a,b present the measured and simulated transmittance spectra of the fabricated MM, respectively. The measured transmittance of the MM shows a dual-band resonance at 4.2 THz (or 140 cm^−1^, called M1, low frequency) and 5.8 THz (or 194 cm^−1^, called M2, high frequency). In a dual-band resonance of a metal–insulator–metal trilayered MM disk, the low-frequency peak is typically attributed to the magnetic dipole resonance, and the high-frequency peak is attributed to the electric dipole resonance^23^. Here, after varying the simulation conditions, we did not observe such a clear distinction because both resonances in the MM have both magnetic dipole and electric dipole resonant properties. In fact, the periodic Ag–Si–Ag trilayered structure exhibits a strong magnetic resonance in the high-frequency mode, M2. The low-frequency mode, M1, also appears as a weak magnetic resonance, which might originate from coupling between the photonic and magnetic dipole resonances because of the subwavelength-scale periodicity (20 μm, on the order of the observed wavelength, ~70 μm, supported by a Si layer with a refractive index of 3.4). The discrepancy of the peak shape between the simulated and measured results is due to differences in the dielectric constant and structural morphology of the Si layers used in the simulation model and fabricated structure. An additional assignment in this simulation result might originate from the geometry of the MM. In this work, the Ag–Si–Ag trilayered structure was elevated from the sapphire substrate, and this elevation provided a longer interaction range of the electromagnetic field among them (along the surface plane) because the dielectric screening of the Si is significantly reduced^24^. Figure 2c shows the results of further simulations of the electromagnetic field distribution, which were performed to obtain more insight into the relationship between these two modes. As observed, the high-frequency mode, M2, exhibits exhibited strong electromagnetic field enhancement, where the E field is located at the edges of the Ag slabs with an enhancement factor as high as 19, whereas the H field is strongly confined inside the Si layer stack, with an enhancement factor of 23.3. This result indicates that the strong H field is induced by coupling between antiparallel oscillating electric dipoles at the top and bottom Ag slabs. In contrast, the low-frequency mode, M1, which is attributed to coupling between the photonic and magnetic dipole resonances in the periodic metal–insulator–metal trilayered MM disk, shows weaker electromagnetic field enhancement compared to the high-frequency mode, M2; the electric field and magnetic field enhancement factors are 8.2 and 9.7, respectively.

### Sensing characteristics

**Figure 3:**
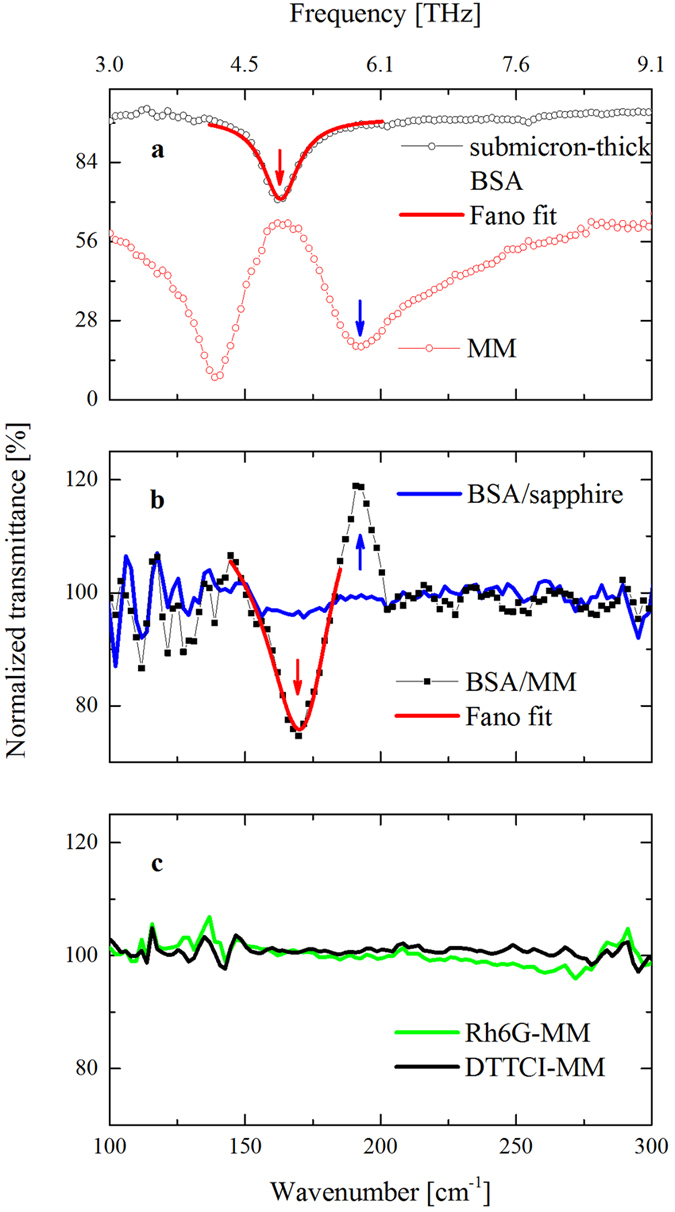
(**a**) Normalized transmittance spectrum of a submicron-thick BSA protein layer (black circles), which was measured before the experiments; the signal strength shows a transmittance of approximately 25%. The spectrum was plotted with the transmittance of the MM sample (red circles) to show the matching of the protein signal and MM resonance. Red line shows the Fano fit for the signal of the submicron-thick BSA. (**b**) Normalized transmittance spectra of an ultrathin layer of BSA molecules absorbed on the MM sample and a reference sapphire substrate. (**c**) Spectra of the organic molecules DTTCI and Rh6G measured in conditions similar to those used for the BSA sample. For details, see the text.

Figure 3 presents the results of BSA protein sensing using our MM. As previously stated, before the experiment, submicron-thick bulky samples of organic molecules [BSA, 3,3′-diethylthiatricarbocyanine iodide (DTTCI), and Rhodium 6G (Rh6G)] were measured. The bulk molecular layers were prepared by dropping solutions of the corresponding molecules onto the substrates and drying them in a stream of N_2_ gas. Between 50 and 2000 cm^−1^, BSA is the only molecule to display a vibration signal, which is located at 4.8 THz, as shown in Fig. 3a. The spectral position and features of the BSA signal presented here is close to those described in an earlier report by Yoneyama *et al*.^25^. However, the absorption spectra of BSA in the THz may vary depending on the preparation (treatment temperature) of the films as well as the molecule’s conformation at the interface and the wettability on specific substrates^26^,^27^. The BSA spectrum was plotted with the MM spectrum to emphasize the spectral matching of the MM resonance and the target molecules’ signal. Although the initial purpose to obtain perfect matching of the M1 mode to the vibration signal of BSA) was not realized, the discrepancy between the simulation and experiments (displayed in Fig. 2) might be small enough to neglect. The BSA signal is located between the two modes of the MM and at a higher energy than the M1 mode of the MM.

Figure 3b shows the enhancement of the BSA signals caused by the presence of the MM amplifier. An ultrathin layer of BSA molecules on the sapphire substrate displays a weak, broad, and negligibly small signal at the frequency of interest. In contrast, an ultrathin layer of BSA on the MM sample exhibits two features. The vibration signal of bulky BSA is strongly enhanced at a nearly identical frequency. The recorded signal strength is almost comparable to that of the bulky sample (~25%). It is worth noting that a BSA layer of this thickness can be considered to approximate a single layer because the BSA molecules were chemically absorbed on the MM for 14 h and subsequently rinsed with distilled water, following a procedure in the literature^28^. The M2 mode of the MM sample displays a tendency toward reverse absorption while maintaining the same energy, which can be understood fairly well because the BSA molecules covered the top Ag surface and changed the permeability of the entire system.

In Fig. 3b, there is no prominent change in the M1 mode, which indicates the insensitivity of our sample design to changes in the dielectric function of the absorbed molecules. This result demonstrates that our design differs from that of splitting resonators, which exhibit ultrasensitive detection because there are molecules in the split rings.

To confirm the sensitivity of the MM sample, DTTCI and Rh6G were also deposited on the MM sample and measured under similar conditions. The results (Fig. 3c) show nearly 100% transmittance in the entire range, and there is no signal for either DTTCI or Rh6G. The reason might be that the vibrational frequency of these small molecules is far from the resonance frequency of the designed MM. In addition, the resonance frequency of the fabricated MM is rather insensitive to changes in the dielectric function. Because minor nanoscale structural defects do not significantly change the optical properties of the micrometer-scale MM, the existence of defects on the MM surface should not be the main reason for either the observation of the BSA signals or the inactivation of DTTCI and Rh6G under THz radiation^29^,^30^. Nevertheless, this observation is consistent with the absorption measurements of the bulk samples because BSA is the only molecule that exhibits vibrational signals between 50 and 2000 cm^−1^.

The selective sensing by the MM of BSA, whose signal is located at the MM resonance, is intriguing because its mechanism is similar to that of plasmonic sensing at optical frequencies^6^,^7^. However, metals are considered perfect conductors in the THz range because of the limited penetration depth of radiation. Our simulation clarified that the electromagnetic field enhancement at the metal structure was larger in the E-field than in the H-field. However, compared to the optical frequencies (visible and near- to mid-IR), the THz range has a smaller enhancement factor. The observed enhancement of the vibrational signal of BSA in this case is assumed to be the result of coupling between the molecular vibration and the electromagnetic resonance of the MM^6^,^31^,^32^,^33^.

In addition, the spectral shape of BSA reveals enhancement of the BSA signal but not the spectral shift of the MM sample resulting from the dielectric coverage, which is usually observed in sensing measurement. Figure 3b shows that the resonant M1 peak disappears in the normalized transmittance spectrum, whereas the M2 peak remains at ~185 cm^−1^ after the MM is covered with BSA. Thus, the M1 peak does not change because the dielectric property between the two Ag slabs was not modified after BSA absorption. For the M2 peak, if a peak shift had occurred because of dielectric modification of the surrounding medium, it would have occurred toward a higher energy (higher wavenumber) and created an asymmetric feature toward the higher-energy side instead of the low-energy side. More conclusively, the dielectric-function-induced spectral shift was not observed for DTTCI and Rh6G coverage, as observed in Fig. 3c, which helps us rule out the sensing mechanism based on dielectric modification.

The spectral line shape of BSA for the bulk materials and ultrathin layer on the MM is an intriguing issue to be analyzed in the context of the Fano resonance, which stems from interference between the discrete vibration signal of BSA and the broadband resonance of the MM^34^,^35^,^36^. The Fano resonance indicates the strength of this coupling and is described as


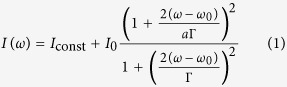


where *I*_const_ and *I*_0_ are dimensionless parameters, *ω*_0_ is the plasmon resonance of the MM, Γ is the spectral linewidth, and *a* is an asymmetric factor that reflects the coupling strength of the two oscillators. The spectra of BSA in the bulk state and on the MM surface were fitted (Fig. 3a,b). The fitted asymmetric factors were *a*_bulk_ = 6.3 × 10^−6^ and *a*_MM_ = 0.17 ± 0.04 for the bulk and ultrathin layer, respectively. The strong distortion of the spectral shape because of Fano coupling indicates that interference between the BSA signal and the resonant signal of the MM occurred and resulted in a huge enhancement of the BSA signal strength.

To further understand the sensing mechanism of the MM, we simulated the dependence of the normalized transmittance spectrum of the BSA-covered MM sample on the vibrational signal of artificial BSA and its damping factor. We introduced an oscillator into the dielectric function of sapphire to describe the absorption feature and used it as an “artificial dielectric function” for molecules (sapphire does not display any absorption feature in the range of interest). We investigated the dependence of the normalized transmittance spectra of the MM in the presence of molecules on the resonant frequency *ω*_0_ and damping factor *δ* of the molecules.

**Figure 4:**
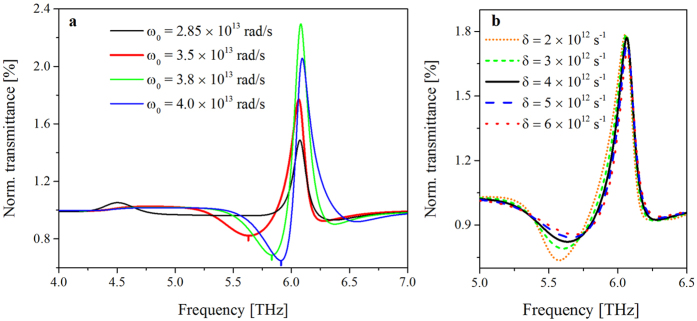
Dependence of the simulated normalized transmittance on the (**a**) resonance frequency and (**b**) damping factor of BSA.

As shown in Fig. 4a, the simulation was performed using a fixed damping constant of the molecules, *δ* = 4 × 10^12^ s^−1^, whereas *ω*_0_ was varied from the low-frequency resonance of the MM, which corresponded to *ω*_0_ = 2.85× 10^13^ rad/s (≈4.5 THz), to above the high-frequency resonance of the MM, which corresponded to *ω*_0_ = 3.8 × 10^13^ rad/s (≈6.1 THz). The normalized transmittance is obviously enhanced when *ω*_0_ approaches the high-frequency magnetic resonance of the MM. This enhancement is maximized at the high-frequency resonance of the MM. Then, the normalized transmittance decreases when *ω*_0_ exceeds this frequency. The antiresonant-like feature of the simulation is obtained in the low-energy part of the resonant peak, which is consistent with the experimental data. Hence, the BSA vibration signal and high-frequency magnetic mode M2 are in a coupling regime, which is reflected by the Fano-like resonance^6^,^34^,^35^. In the simulations, similar behavior is obtained for the low-frequency resonant mode, although it is much less effective than that for the high-frequency resonant mode. This result might occur because the electromagnetic field enhancement factors at the low-frequency resonance, M1, were lower than those at the high-frequency resonance, M2 (Fig. 2c). In Fig. 4b, the dependence of the normalized transmittance on the damping factor of the molecules was simulated by changing *δ* while maintaining *ω*_0_ = 3.5 × 10^13^ rad/s. The damping factor affects mainly the signal strength of the molecules but does not severely affect the resonant frequency. Although varying the damping factor of the BSA molecules is outside the scope of the current work, the simulated result may help to elucidate the sensing of proteins with various degrees of crystallinity because the damping factor may be closely related to the preparation of protein crystals.

## Discussion

In summary, we demonstrated that a THz MM with an appropriate design exhibits a molecular sensing ability similar to that of surface-enhanced spectroscopies at optical frequencies (SERS and SEIRA). We show that it can enhance the vibrational fingerprints of organic molecules at the MM resonant frequency. This result indicates that coupling and interference between the electromagnetic resonances in the MM and the molecular vibration are the main mechanisms of signal detection. It is worth noting that although the detection mechanism was thoroughly investigated, the detection limit of this new sensing method was not yet fully examined. To address the detection limit, quantitative evaluation of the coverage of BSA (or other proteins) will be performed in the future^6^,^7^,^32^. The present MM-enhanced spectroscopy may be of special use because it offers a simple approach to nondestructively detecting large biomolecules, which creates a new avenue for adopting THz sensing for industrial applications.

## Methods

The Ag–Si–Ag trilayered structure was fabricated on a 1 cm × 1 cm sapphire substrate using a two-step, standard photolithography process. The thicknesses of the trilayered disks from bottom to top were 0.5, 3, and 0.2 μm, respectively.

The simulation was performed using a finite-integration package (CST Microwave Studio). The MM structure consisted of three layers, which were supported by a sapphire substrate. The top and bottom layers were metallic square arrays, whereas the middle layer was a square array of a dielectric. In the simulation, the electrical conductivity of silver was 6.3 × 10^7^ S/m, and the permittivities of silicon and sapphire were 12 and 9, respectively^37^,^38^. The incident electromagnetic wave propagation was normal to the MM surface. The electric and magnetic fields were polarized along the sides of the square arrays. The amplitudes of the output fields were normalized to their incident amplitudes.

To simulate the effect of BSA coverage, a thin material layer was placed on top of the MM structure. Because the dielectric function of BSA is not available in the literature, we intentionally introduced an oscillator representing the absorption feature into the dielectric function of sapphire and used it as an alternative dielectric function of the absorbed molecule layer. This material was simulated using the Lorentz model so that it could exhibit a similar resonance response to that of actual BSA.


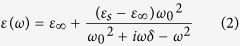


where *ε*_∞_ = 1, *ε*_*s*_ = 2, and *ω*_0_ and *δ* are the resonant frequency and damping factor of the oscillator, respectively.

The morphology of the fabricated MM sample was examined using a Hitachi SEM S4800 microscope at an accelerating voltage of 10 kV. The optical property of the Ag–Si–Ag MM was measured in the transmittance geometry using a Fourier transform infrared (FTIR) spectrometer (Thermo Nicolet NEXUS 670 FT-IR) with a far-IR solid-substrate beam splitter and a polyethylene-windowed DTGS detector. The FTIR spectrometer provides a spectral range that covers the far-IR region in the frequency range of 50–2000 cm^−1^ (1.5–60 THz). A 1 cm × 1 cm blank sapphire substrate was used as a reference for the measurement. The normalized transmission measurement is defined as a normalization of the transmittance of a sample over the substrate. To avoid machine drift over time, those measurements were performed separately by normalizing to the empty beam in a purged medium. For the biochemical sensing measurement in the far-IR region, BSA, which is expected to have a vibration at 165 cm^−1^, was used as a target molecule. The MM sample and a blank sapphire substrate were dipped into the BSA solution (300 mM in water) for 14 h. Then, the BSA-coated samples were rinsed in distilled water and finally dried in a stream of nitrogen gas. The submicron-thick BSA sample was obtained by drop-casting 10 μl of a 300 mM BSA solution on the reference substrate. All optical measurements were performed at room temperature; before the experiment, the FTIR chamber was purged by dried nitrogen, and its spectral stability was examined.

## Additional Information

**How to cite this article**: Bui, T. S. *et al*. Metamaterial-enhanced vibrational absorption spectroscopy for the detection of protein molecules. *Sci. Rep.* **6**, 32123; doi: 10.1038/srep32123 (2016).
